# Sleep Across the Lifespan: A Neurobehavioral Perspective

**DOI:** 10.1007/s40675-025-00322-2

**Published:** 2025-02-05

**Authors:** Katharine C. Simon, Chelsea Cadle, Alessandra E. Shuster, Paola Malerba

**Affiliations:** 1 Department of Pediatrics, School of Medicine, University of California, Irvine, Irvine, USA; 2 Pulmonology Department, Children’s Hospital of Orange County, Orange, USA; 3 Center for Biobehavioral Health, Research Institute at Nationwide Children’s Hospital, Columbus, OH USA; 4 Cognitive Science Department, University of California, Irvine, Irvine, USA; 5 Department of Pediatrics, School of Medicine, The Ohio State University, Columbus, OH USA

**Keywords:** Sleep, Polysomnography, Development, Neurophysiology, Sleep patterns, Sleep behaviors

## Abstract

**Purpose of Review:**

Sleep is dynamic across the lifespan, influenced by brain maturation, neurophysiology, hormones, and cognitive processes. Sleep behaviors influenced by physiological and external factors can also impact sleep health. As sleep plays a mechanistic role in health across the lifespan, understanding when and how to intervene to benefit health is essential.

**Recent Findings:**

Recent research has advanced our understanding of sleep across three domains: patterns, neurophysiology, and behaviors. Highlights include (1) Early childhood nap cessation is thought to relate to medial temporal lobe network maturation and underlie long-term hippocampal-dependent memory development. (2) Chronotype misalignment is a key factor in sleep deficits and social jetlag. (3) Older adult daytime sleep has complex effects on health, at times beneficial while others, potentially maladaptive. (4) Longitudinal sleep oscillation trajectories are starting to be investigated and indicate neurophysiology could be interpreted as indicative of brain maturation in development. (5) In adults, sleep quality and macrostructure trajectories show high variability, emphasizing distinctive traits in shaping sleep and its lifespan trajectories. (6) Neighborhood and socioeconomic factors influence sleep health across all ages. (7) In older adults, associations between loneliness and poor sleep are being unpacked.

**Summary:**

This recent research, while comprehensively describing our current understanding of sleep trajectories across the lifespan, emphasizes the need to expand current approaches to longitudinal measurement studies that cross age-spans. Expanding will enhance our ability to mechanistically determine the temporal and causal relations between the multiple dimensions of sleep (i.e., patterns, behaviors, and physiology) and outcomes in sleep health.

## Introduction

Sleep patterns and neurophysiology change dynamically across the lifespan [1]. Various physiological and mental health factors can modulate neurophysiology, alter sleep patterns, and influence behaviors that can either enhance or impede quality sleep [2–5]. Despite a clear understanding that poor sleep affects health, and health, in turn, affects sleep–and that these effects can compound over time [6, 7]—we still lack a detailed mechanistic understanding of how sleep and health interact dynamically across the lifespan. This knowledge gap underscores the need to independently and interactionally examine sleep across multiple dimensions, as defined by Buysse (2014) [8]. Age-associated changes in each of these dimensions parallel underlying brain and body maturation and subsequent degeneration trajectories across the lifespan [9, 10]. In this review, we focus on three key dimensions of sleep: patterns, neurophysiology, and behavior. By examining each dimension across distinct periods of the lifespan, we aim to highlight the interplay and concurrence of changes within and across these domains.

**Fig. 1:**
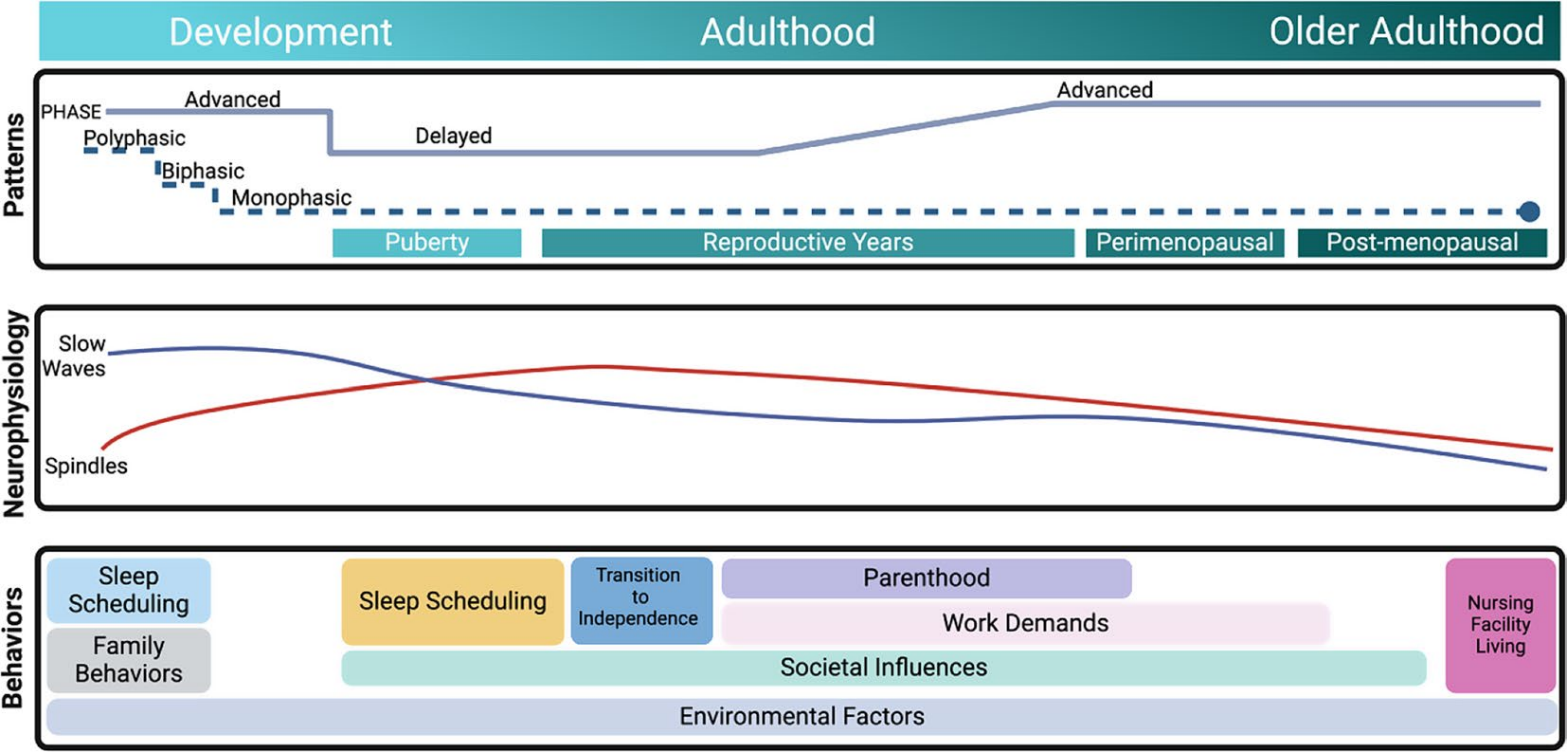
Sleep patterns, neurophysiology, and factors that impact behaviors are presented across the lifespan from development to older adulthood [14]. Sleep patterns include the natural cycle of wake and sleep over 24-hours, regulated by the circadian rhythm and neurobiological systems responsible for sleep/wake transitions. This panel demonstrates changes to circadian rhythms and consolidating sleep patterns with respect to hormone profiles and aging. Sleep neurophysiology includes the macro and microstructure characteristics of sleep across a night. Shown are the general changes of specific NREM sleep oscillations, slow wave activity and spindle density, across the lifespan. The last panel shows external factors that influence sleep behaviors and resulting quality of sleep across the lifespan

In this review of literature from the last five years, we define sleep patterns as the natural cycle of wake and sleep over 24-hours, regulated by the circadian rhythm and neurobiological systems responsible for sleep/wake transitions. We define sleep neurophysiology as the macro and microstructure characteristics of sleep across a night, such as the organization of sleep stages and specific electrophysiological events. Finally, we define sleep behaviors as the actions individuals take that promote or impair the quality of their sleep. Behaviors can be distinguished from patterns, as patterns refer to endogenous factors impacting sleep, while behaviors refer to external actions an individual takes that influence sleep (e.g., sleep scheduling) and external factors that influence sleep (e.g., neighborhood safety). While external factors can also impact patterns, we focus on the endogenously driven components, such as circadian rhythmicity in discussion of sleep patterns. Figure 1 offers an overarching graphical abstract of the review’s organization.

Literature on age-associated trajectories and interactions across sleep dimensions is often built on cross-sectional or two-wave longitudinal studies, limiting our ability to examine the temporal ordering nuances between sleep and health. Longitudinal intraindividual measurement studies that cross age-spans are fewer, but provide a more complete understanding of the changing relationships over time [11–13]. These types of studies further allow us to generate causal conclusions of when and how sleep influences health outcomes, and are essential to the identification of malleable factors, including the most effective timing for intervention across the lifespan. Since determining specific targets and timing windows are essential to the development of effective sleep-based interventions, a comprehensive description of the interrelations between sleep patterns, neurophysiology and behaviors across the human lifespan is required.

## Sleep Patterns

We define sleep patterns as the natural cycles of wake and sleep across a 24-hour period, which are governed by the circadian rhythm and neurobiological systems responsible for regulating sleep/wake transitions. Age-associated changes in sleep duration and timing are present across the lifespan, and pattern alterations are linked to underlying neurobiological and physical maturation, and environmental factors.

### Development (Infancy to Late Adolescence)

Sleep patterns show the most dynamic age-related changes in duration and timing during the period from infancy through late adolescence. Initially polyphasic, newborn sleep occurs over multiple distributed bouts, typically up to 18 h of sleep across a 24-hour period [15]. Over the first few months, newborns’ sleep consolidates into one longer overnight period and multiple daytime naps, reflective of the development of circadian rhythmicity [16]. Evidence of the exact onset of circadian rhythmicity in the first year of life is mixed, but sleep-associated melatonin and cortisol hormone secretion are reported at approximately 3 months of age, with continued maturation over the first year of life [17]. Daytime napping frequency continues to reduce into longer, but fewer daytime naps and an extended overnight sleep [16, 18]. By toddlerhood, sleep is typically biphasic [16, 19], which is influenced by brain maturation and the rate at which homeostatic sleep pressure builds [20]. Napping cessation often occurs between 3 and 5 years in typically developing children, and linked to a number of genetic, cultural, environmental, familial, and social-demographic factors [16, 21]. According to one recently proposed theory, the timing of nap cessation is linked with maturation of the medial temporal lobe network; when this network is mature, the rate of homeostatic sleep pressure accumulation decreases, and the brain can extend periods of wakefulness [22]. Behaviorally, this is linked to enhanced long-term hippocampal-dependent memory formation [22].

Sleep duration trajectories have been demonstrated from infancy through childhood [23–26], though most studies have been conducted in Western, Caucasian populations. A recent study by Tham et al. examined sleep trajectories as reported by parents on validated sleep questionnaires in Asian infants from 3 months to 4.5 years, identifying four sleep pattern groups that included both daytime and nocturnal sleep [24]. Infants in the long-variable and long-consistent groups had the greatest sleep duration in infancy, with the long-variable group maintaining the greatest duration across the first two years. Both groups eventually declined to approximately 11 h of sleep, matching the moderate-consistent group, which maintained steady total sleep duration from infancy through 4.5 years. Conversely, the short-variable group began with the lowest sleep duration in infancy, and increased as toddlers, but remained significantly lower in total duration than the long-sleep groups at 18 and 24 months. By age 4, total overnight sleep duration converged across all groups. However, the short-variable trajectory group continued to exhibit the highest variability in sleep patterns [24]. Studies at specific timepoints in the first two years of life highlight the importance of sleep in early cognitive development. For example, better sleep efficiency at 10 months is associated with higher mental developmental index scores on the Bayley Scales for Infant and Toddler Development [27]. Additionally, daytime naps at 6.5 and 15 months have been shown to support novel language acquisition [28, 29]. Conversely, short sleep duration in early life is linked to poorer behavior [30, 31], mood symptoms [32–34], and worse cognitive outcomes [25, 35] in toddlerhood and childhood. Since early sleep patterns are strongly influenced by parenting behaviors, such as sleep scheduling and boundary setting, behavioral interventions are one way to effectively support high-quality sleep [36].

During childhood (6 to 11 years) sleep patterns remain relatively stable, primarily influenced by school schedules, extracurricular activities, and parental bedtime enforcement [36]. In early childhood, sleep patterns are generally biased towards a morningness circadian profile [37]. Furthermore, differences between weekday and weekend sleep timing and duration remain low [37]. As children age and undergo pubertal maturation, their circadian phase shifts later [37, 38]. Pubertal sex hormones typically begin around ages 9 to 10 in girls and around 10 to 12 years in boys [39]. These hormonal changes are linked to ongoing brain maturation and coincide with later sleep onset, longer sleep onset latency, and shorter total sleep duration [40]. The biological systems driving this phase delay include reduced homeostatic sleep pressure, reduced nocturnal plasma melatonin levels, and delayed melatonin onset [see review [41]]. Interestingly, neither circadian period [42] nor sensitivity to evening light appear to be driving factors of this age-associated phase delay [43]. However, further external psychosocial factors, such as increasing academic work, extracurricular activities, independence, and social relationships, contribute to the mismatch between societal timing expectations and intrinsic circadian profiles [42]. A consequence of this mismatch is a propensity for insufficient sleep, evident in the difference between sleep duration on weekdays compared to weekends [44]. This delayed circadian profile is consistent across cultures, with adolescents often having later bedtimes and greater sleep restriction [45]. Furthermore, greater intraindividual sleep variability, specifically increased bedtime variability and less total duration during adolescence has been associated with worse emotional, cognitive, behavioral, and psychological health [46] and negatively affects brain maturation and neural circuitry [47].

### Adulthood

During emerging adulthood (18 to 25 years), the recommended sleep duration per night is 7–9 h, and the nocturnal distribution of sleep remains relatively stable [48]. Despite this recommendation, the transition from adolescence to adulthood continues to be accompanied by sleep deficiency [36] and intraindividual night-to-night sleep pattern variability [36, 49]. Factors influencing these changes may include increased independence, less structured routine, and fluctuations in daily life activities [36]. There are mixed findings on whether living arrangements, specifically moving to university housing, influence sleep variability [49, 50]. However, in one study using actigraphy and sleep diaries, the three-day standard deviation mean for college students’ sleep duration was equivalent to just over 90 min [51]. In a separate study, those with more significant sleep variability during college appeared to be less likely to graduate with a college degree suggesting that maintaining consistent sleep patterns may be one important factor for academic success [52].

The transition to adulthood is often accompanied by a shift to an earlier chronotype profile, although this is found to differ between biological sexes. On average, between ages 20 to 50 years, men show greater evening preference than women on average [21], and these sex differences disappear after the age of 50, coinciding with menopause in women [22]. Some research suggests that this chronotype difference disappears as women become more evening-oriented, while other research shows that women become more morning-oriented during the menopause transition [53–55]. Thus, more research is needed to understand the dynamics of how chronotypes change with age across sexes. Importantly, several environmental factors specific to the young and middle adult life stage, such as parental responsibilities and atypical work hours, can lead to undesired circadian misalignment. Inconsistent circadian rhythm regularity is an independent predictor of adverse health outcomes, diminished work performance, lower ratings of subjective sleep quality, declines in mood, and heightened risk for depression [56]. These associations are thought to arise from decreased circadian synchronization across organ and tissue systems, disorganized patterns of cellular and organismal stress, and weekday-to-weekend fluctuations in sleep timing that may induce repetitive episodes of circadian disruption and occasional sleep insufficiency [56]. Von Gall and colleagues (2024) recently conducted a real-world longitudinal study to understand the relationship between circadian rhythms and sleep composition during social timing conditions (workdays) and biological timing conditions (days off) [57]. On workdays, the midpoint of sleep was significantly earlier, and sleep duration was significantly shorter, compared to free days [57]. Further, those with a later chronotype had greater social jet lag (i.e., misalignment between an individual’s internal circadian rhythm and external social schedule) and sleep loss.

Implicated as a primary source of interference in multiple sleep-health domains, new parents encounter formidable challenges to recommended sleep duration, regularity, and timing. Pregnancy, childbirth, and the postpartum period are associated with significant sleep disruption. Reports of altered sleep duration, quality, and patterns occur in women at all stages of pregnancy, with up to 78% of women reporting sleep disturbances during the third trimester [58]. Sleep disturbances in pregnant women are associated with numerous medical conditions, including pre-eclampsia, gestational hypertension, gestational diabetes mellitus, cesarean section, preterm birth, and stillbirth, compared to those without disturbed sleep [59]. Sleep disruption during the postpartum period begins with delivery and ends for most women approximately six to twelve months later, when the infant is sleeping through the night [60]. Recent evidence links postpartum sleep disturbances with poor outcomes, including postnatal maternal depression and offspring impaired cognitive development in early childhood [59, 61]. King and colleagues (2020) found that poor sleep continuity in mothers with postpartum was associated with the inability to sustain sensitivity toward infants during a home-based 10-minute free play interaction [62]. Indeed, insomnia diagnosis in postpartum mothers is significantly correlated with increased household chaos and with lower self-efficacy and satisfaction in the parenting role, even when controlling for socioeconomic status, depression, and relationship status [63].

For females, middle adulthood is characterized by the transition through menopause, which starts on average between the ages of 46–55 years and is a multi-year process during which sex hormones decline and ovulation ceases [64, 65]. Reports indicate that 40–60% of women in this perimenopausal to postmenopausal age range have a high prevalence of sleep complaints [66]. Moreover, the natural decline in estrogen at this stage is associated with declines in sleep quality, efficiency, and continuity [67]. While women report more sleep complaints, research shows that men have a greater degree of objective sleep decline as they progress through adulthood including greater decreases to time spent in slow wave sleep and increases to time in lighter NREM stages 1 and 2 [1, 68]. However, multiple studies have found differences between subjective (i.e. self-reported sleep quality) and objective sleep metrics such as TST, SOL, and time in each sleep stage, suggesting that these metrics may reflect different aspects of sleep [69, 70]. In women, decreasing estrogen levels amplify vasomotor symptoms, leading to nocturnal sweating and hot flashes, further compromising sleep quality [71]. For women, increased sleep disturbances after menopause are associated with higher likelihood of inflammation, cardiovascular, and metabolic diseases [66]. These findings underscore the potential health risks associated with sleep pattern changes in midlife, particularly for women.

### Older Adults

Sleep patterns undergo significant changes in older adulthood, some irrespective of the medical comorbidities often associated with aging [72]. These alterations include tendencies towards earlier sleep onset, reduced total sleep duration, struggles with sleep maintenance, and heightened sleep fragmentation [72, 73]. Adjustments in sleep-wake timings are associated with circadian phase advance, marked by earlier onset of evening sleepiness and earlier morning awakening [72]. On average, this earlier shift in sleep timing amounts to approximately one hour and is believed to mirror biological alterations in the circadian rhythm, such as an overall decline in melatonin levels, earlier melatonin release, cortisol secretion advance, and a diminished range of core body temperature fluctuation throughout the day [72]. Recent studies further provide evidence that there is a progressive decline in the amplitude of the circadian oscillation and a reduction in the circadian period, signifying a weakening of rhythmicity with advancing age [74]. Mechanistically, these circadian alterations are associated with a decline in the functionality of the suprachiasmatic nucleus, a structure responsible for regulating the circadian clock [75]. Combined with circadian factors, age-related reductions in sleep homeostasis resulting in decreased accumulation of sleep pressure also contribute to shifts in sleep timing [76]. Overall, the earlier shift in sleep is thought to be a part of normal aging as it is present in otherwise healthy older individuals [77]. However, it can contribute to greater deviations from a regular sleep schedule, such as the case where older adults unintentionally doze off in the early evening, take a nap, then wake up and get ready for bed only to have difficulty falling and staying asleep [78]. These cases may contribute to secondary complaints of insomnia, which is associated with mechanistic sleep alterations such as deficits in slow wave activity in older adults [78, 79].

Once asleep, older adults experience increased sleep fragmentation and difficulty maintaining sleep, contributing to less total sleep. The risk for disordered sleep increases with age, and the increased prevalence of conditions such as insomnia, restless leg syndrome, and sleep apnea directly contribute to more frequent nighttime awakenings [3]. Beyond sleep disorders, general health changes also affect sleep. Age-related changes in bladder control lead to more frequent awakenings during the night [80, 81]. Additionally, the majority of older adults have comorbidities, and over 90% of individuals over 65 years take prescription medications, many of which exacerbate sleep disturbances [82]. Over 30% of older adults take five or more medications [80] for medical issues such as cardiovascular disease, hypertension, respiratory issues, and dementias, all of which are linked to disturbed sleep [83]. Disordered sleep has also been shown to worsen symptom severity for many of these diseases, contributing to a positive feedback loop of adverse health outcomes and worse sleep [84–86].

Daytime sleep habits (e.g., napping) are also known to change in older adults. Older adults report increased diurnal napping compared to their younger and middle-aged counterparts across cultures [87, 88]. One theory is that increased napping is compensating for decreased nighttime sleep and is appropriate for normal, healthy aging [77]. However, napping in older adults has also been linked with the exacerbation of chronic health conditions [87, 88]. Indeed, longer naps of over 1.5 h are associated with worse outcomes for cardiovascular health, diabetes, cognitive function, and increased mortality in older adults [88]. Yet, it is not certain if there is a causal relationship between maladaptive napping and worse health outcomes or if these longer naps are merely symptomatic of overall health issues. Other studies have investigated the impact of nap interventions on cognitive function in older adults and demonstrated that intentional naps benefited cognition across numerous domains [reviewed in [72]]. More data is needed to disentangle how different types of naps relate to healthy, normal aging outcomes vs. age-related pathologies.

### Summary

Recent research using longitudinal approaches has highlighted exciting insights into sleep pattern trajectories both within and across individuals. However, the majority of studies of age-associated sleep patterns rely on cross sectional or limited longitudinal designs, which fail to examine pattern changes within an individual or across age periods (e.g., adolescence through middle adulthood). Our understanding of how these associations evolve over time and our ability to determine directionality remain limited. For example, the current literature has yet to resolve key features of sleep patterns, such as the role of napping in early or later life, and more broadly, how sleep patterns may reflect potential health conditions or contribute to the development or progression of chronic disease. At the level of sleep patterns, it is crucial to understand the interplay between circadian aspects of physiology and the organization of body physiology across the lifespan (e.g., neural reorganization, hormonal expression, stress levels) to identify optimal intervention times that have the highest likelihood for positive impact. Future research can benefit from multi-wave longitudinal, measurement burst designs that capture the temporal progression and interplay among sleep, health, cognition, physiology, and neurobiology. Studies employing such multi-wave longitudinal designs offer valuable insights into changes over time. However, these types of studies can face challenges, particularly with cost, time, and participant retention. To address these issues, careful planning is essential, including the implementation of regular follow-ups, appropriate compensation, and flexible data collection methods (e.g. components that can be completed remotely in lieu of in-person assessments). Additionally, statistical methods that handle missing data effectively, such as mixed-effects models, can allow researchers to include all collected data, even for participants who do not fully complete the study. Accounting for these factors during the planning phase is critical for ensuring the success of these types of studies, maximizing their potential to provide meaningful insights into the complex temporal dynamics of sleep, health, and the biological processes underlying these changes.

## Sleep Neurophysiology

We describe sleep neurophysiology as organized in a macrostructure, referring to the overall organization of sleep stages across a night of sleep, and a microstructure, referring to electrographic events that are typical of specific sleep stages, such as slow wave activity (0.5 to 4 Hz) and spindles (12–15 Hz).

### Development

Across development, brain activity in NREM and REM sleep mirrors underlying structural and functional brain development and reorganization [9, 89]. Maturational patterns can be seen from birth through late adolescence in features of NREM and REM sleep, specifically K-complexes (i.e., single, large slow waves), slow wave activity (SWA, 0.5–4 Hz, a spectral measure including slow oscillation activity), spindles (12 to 15 Hz, bursts of thalamocortical activity), and power in the delta (1–4 Hz) and theta (5–8 Hz) bands [9, 90, 91]. K-complexes are commonly observed to emerge between 3 and 6 months, with increased consistency by childhood [92]. During the early months of development, NREM theta power declines over a sleep episode, suggesting that theta may be a developmental proxy for homeostatic sleep pressure [93]. Mirroring neural structural and functional maturation and connectivity, the peak distribution of SWA travels across a posterior to anterior axis during development, with SWA reaching maximal amplitude shortly before puberty and decreasing during adolescence [94–97]. SWA spatial organization shows highest density in central locations in childhood and enhanced frontal presence in adolescence, progressively tracking development of the frontal lobe [94, 97, 98]. Spindle maturation also tracks development, increasing in rate and concordance across hemispheres from toddlerhood through adolescence [99, 100]. While within-subject longitudinal data on spindle maturation is scarce, Feinberg and colleagues tracked multiple cohorts of youth from 6 to 18 years, demonstrating maturational changes in spindle characteristics including within-subject increase in spindle frequency from childhood to adolescence, and decline in spindle amplitude in adolescence [11, 101, 102]. Spatially, spindle density reaches a relative maximum with equal distribution across frontal, central, and parietal areas during adolescence [103]. In childhood and adolescence, spindle activity is more concentrated in frontal regions during primary school age with centro-parietal faster spindles emerging and becoming more prominent in late adolescence [99]. Over the course of development, the rate of coupling of spindles to slow oscillations (SOs, 0.5–1.5 Hz, oscillatory events that contribute to SWA [98]) increases at central locations [104]. This increased coordination of spindle-SO activity in central regions is thought to be secondary to the emergence of relatively faster spindles in this area. Research has explored the cognitive ties of neurophysiological maturation of NREM sleep oscillations across development, with a meta-analysis showing that spindle properties in youth 8–22 (in particular sigma power) are related positively to fluid IQ, measures of working memory and executive functions, and motor speed (e.g. finger tapping task), but not to overall IQ [105], also supported by more recent work [106] showing a positive relation between sigma power and reaction time in typical 9–12 years old. Similarly, organization of SWA is directly linked to cortical myelination [95] and often interpreted as an indicator of brain maturation, with data showing that the relation between visuomotor training and localized expression of SWA is found across development, and is strongest in children [107]. Overall, these observed correlations between NREM sleep neurophysiology and cognitive measures suggest that sigma power and SWA could be interpreted as indicators of brain maturation, with sleep EEG signatures of more ‘maturity’ relating to better cognitive performances across domains. Of note, within this interpretation, the relation of spindle/sigma to brain maturation would not be uniform across development. For spindles in young children, a lower density could be indicative of brain maturation [108, 109], while in later years, when faster central sigma/spindle activity emerges, the maturation indicator would be switching to increased sigma power. In contrast to NREM sleep, the developmental changes in REM neurophysiology have been less studied. REM sleep amount is most prevalent at birth and declines from infancy through early childhood [9]. During infancy, general spectral power is known to increase in both sleep stages, but remains proportionally lower in REM than NREM [93]. Spatially, REM theta activity peak is predominant in the posterior regions around one year and frontal regions around two years [110]. Longitudinal studies tracking intraindividual changes between middle childhood and late adolescence show REM delta wave rate and power decline linearly, with the greatest rate of decline around age 6 [11]. Although the functional role for REM sleep across development remains unclear, current theories suggest a role for REM in cortical plasticity, with recent work linking myoclonic muscle twitches, a prominent feature of infant REM sleep, to sensorimotor system cortical development [111]. For a recent comprehensive review of the relation between sleep and cognition across development, see review [112]].

### Adulthood

Subtle shifts in the macro- and microstructure of sleep neurophysiology accompany the transition to adulthood. Cross-sectional studies on adult cohorts report an age-related decline in sleep NREM spectral power, including delta, theta, and sigma bands [113]. Females have higher central sigma and delta band powers during deep sleep throughout adulthood from ages 20 to 75, possibly reflecting biological and hormonal influences [114]. Compared to younger adults, older adults show decreased delta power and increased sigma power during REM [115]. In young adults, recent research has attempted to determine the spatio-temporal profiles and organization of sleep Sos and spindles [116, 117]. Malerba et al. (2019) found three clusters of SOs, Global, Local and Frontal SOs, respectively, with each cluster differing in amplitude, spatio-temporal profile, and relation to spindles [118]. Only Global SOs had traveling wave profiles, moving anteriorly to posteriorly, larger amplitudes at frontal electrodes, and higher modulation of spindle amplitude by SOs phase (computed with modulation index [118, 119]). Further, Global SOs orchestrated long-range neural communication (quantified with directed information flow) that correlated with overnight memory improvement [120], suggesting a functional role for different SOs based on their organization on the scalp. For sleep spindles, the most significant changes between ages 21 and 65 include trending decreases in density, amplitude, and duration: Across a night of sleep, the topographic distribution in spindle density shows that young adults display anterior prominence that is not seen in middle-aged and older adult age groups [121]. In central channels, fast sleep spindle density increases from adolescence into young adulthood but then declines thereafter, reaching its peak at age 18 for females and 25 years for males [100]. At ages 50–70, the density of frontal slow spindles is higher in females than males, gradually decreasing thereafter [100].

While cross-sectional studies have suggested an age-related decline in lower-frequency activity during REM sleep, in a longitudinal study monitoring middle-aged adults in their 60s into their older adulthood, Gao & Scullin (2023) observed age-related increases in REM power density across all frequency bands, a finding that challenges previous assumptions [10]. The observed rise in lower-frequency REM activity is potentially linked to the progression of mild cognitive impairment and Alzheimer’s disease in specific individuals. The same work also found that these changes in REM spectral power were prevalently driven by females. While sex differences in longitudinal patterns of sleep microarchitecture are less established, one potential explanation for the difference observed between female and male spectral power is that cortical thinning is associated with reduced delta activity [122], and middle-aged females exhibit thicker cortices in specific brain regions than middle-aged males [123]. They also found over a 5-year period a decline in NREM delta and sigma power and increase in NREM theta power. In contrast to the notion that sleep declines linearly with advancing age, longitudinal trajectories varied considerably across individuals. Results also indicated that the changes in NREM and REM power density within individuals were strongly correlated over a 5-year period suggesting that changes in sleep microstructure are also influenced by individual-level factors (e.g., a chronic health condition), beyond age-associated trends.

### Older Adults

Changes in sleep neurophysiology in older adults (65+) can be tied to age-related cognitive decline [80]. One notable change to macro-architecture is decreased TST, with healthy adults experiencing average reductions of 8–12 min per decade, even though older adults spend more time in bed, thus reflecting decreased sleep efficiency [80]. It is suggested that reduction in TST is not due to reduced sleep need; rather, older adults’ ability to generate sleep is compromised, leading to increased SOL, more WASO, and fewer successive NREM-REM cycles [80]. However, on the contrary, other research posits that older adults indeed have reduced sleep need indicated in part by reduced slow wave sleep in healthy aging [124]. In healthy older adults, shorter durations of NREM-REM cycles (defined as NREM sleep followed by REM sleep with less than 2 min of wake) predict later cognitive decline [125]. In addition to overall sleep duration tending to shorten in advanced age, there are also distinct shifts in the composition of sleep stages, with increased time spent in light sleep (N1) and N2, and decreased time spent in deeper sleep (N3). This decrease in N3 and increase in N2, attributed to cortical thinning [125, 126], may be influenced by the criteria used for scoring sleep stages. According to the current guidelines set by the AASM (American Academy of Sleep Medicine), N3 sleep requires a presence of SWA (75 uV or greater) for over 20% of an epoch. This may contribute to mixed findings on time spent in N3 and cognitive outcomes but does not introduce confounds in findings on specific within-epoch sleep oscillations (described in the next paragraph) [127–129]. There is mixed evidence about changes in REM among older adults, with some studies reporting that REM time stabilizes or increases in older adulthood, while others showing REM time reduction [80, 130]. Between-study discrepancies may be due to sex differences, with some evidence that women have a greater rate of REM decline, as well as differences in healthy vs. abnormal aging, with reduced REM marking increased risk for cognitive impairment [68, 131]. Further investigation is needed to understand the temporal shifts of sleep stages in older adults and to delineate normal age-related shifts from those potentially indicative of pathologic conditions.

Beyond these changes to sleep composition across a night, sleep microstructure changes as well. Older adults, compared to their young adult counterparts, have decreased count and density of K-complexes and spindles, as well as reduced SO amplitude [92, 127]. For spindles, the largest age-related changes are seen in decreased fast spindle density and total integrated spindle activity, a measure of spindle intensity incorporating amplitude, duration, and count, where higher integrated activity reflects overall increase in spindle presence and amplitude [132]. Moreover, the coupling of spindles and SOs reduces with age, and spindles tend to occur earlier in the SO phase in older adults [132]. The time-locking of spindles to the phase of SOs has been shown to promote memory in adults [133] and disrupting SO-spindle phase coupling with optogenetics in mice impairs memory [134]. Separately, SWA is implicated in age-related cognitive decline [127], with reduction of slow waves impairing glymphatic system cleansing [135, 136], which is linked to neurodegenerative diseases [137]. Although slow wave sleep is theorized to support neocortical control of information flow in younger individuals [138, 139], with age the hierarchy reverses, such that spindles are thought to drive slow waves in older adult age groups [140]. Deterioration of slow wave sleep is widely accepted to have a bidirectional relationship to Alzheimer’s dementia (AD): disruption of slow wave sleep increases amyloid-β plaques (Aβ,a hallmark of AD), and experimental increases of Aβ result in decreased and fragmented slow wave sleep [141, 142]. While the connection between REM events in aging and cognitive decline is less understood, recent work shows evidence that lower REM theta power is linked to increased Aβ build up in healthy adults [143]. In younger adults, recent research has identified and linked REM theta burst activity to cognitive processes, with greater burst activity associated with better cognitive performance, but these burst events have yet to be examined in relation to aging and cognitive decline [144]. Future research should examine alterations in sleep microarchitecture among older adults to identify shifts which are indicators of aging alone vs. those which are linked to neurodegenerative disorders and may be targets of interventions.

### Summary

Changes in sleep neurophysiology across the lifespan mirror synaptic reorganization and are directly linked to the cognitive characteristics (desirable or otherwise) of the various life stages. Crucially, while the coarse micro- and macrostructure trajectories of sleep have been mapped, detailed within-subject trajectories that delineate age-related changes in sleep neurophysiology are unknown, as are the links that connect these changes in sleep EEG signatures to factors that are considered mechanistically relevant to shaping such dynamics. As a result, our understanding of the relative causal or modulating role of brain/body physiology, sleep behaviors and sleep patterns on each other, and in relation to health and cognitive outcomes, remains elusive. While all of these three domains of sleep are essential to understanding the role of sleep in health across the lifespan, the authors suggest that sleep neurophysiology is best placed among the three to produce quantifiable early predictors and objective measures of efficacy for sleep-based interventions. This is because the variability of sleep neurophysiology across subsequent sleep nights is much smaller than variability across individuals, and builds on known (if not yet robustly established) trends of differentiation in sleep EEG signatures tied to physical and mental health conditions at all points across the lifespan (e.g., Down Syndrome, Schizophrenia, Duchenne Muscular Dystrophy, AD [145–149].

## Sleep Behaviors

We define sleep behaviors as the actions individuals undertake that either promote or impair their quality of sleep. These behaviors constitute a measurable dimension within the broader scope of sleep health that often reflects adjustments individuals make due to personal, societal, or environmental influences. In the following sections, we describe behaviors that promote sleep health and those that have adverse effects on sleep.

### Development (Infancy to Late Adolescence)

In early development, sleep is influenced by factors such as sleep schedule regularity, bedtime routine consistency, infant temperament, family context, illness, and environmental factors [150–152]. Once circadian rhythmicity develops around 12 weeks, infant sleep/wake patterns typically stabilize and demonstrate increasing nap and nighttime sleep regularity over time [153]. Sleep disturbances during infancy often involve difficulties falling asleep due to sleep-onset associations, limit setting challenges, or night wakings (see [154] or review). Infants with greater negative affect and reactivity are more prone to persistent sleep disturbances, between 6 and 12 months [155]. Beyond temperament, the role of parent-infant attachment has been explored in sleep disturbances, though the findings are mixed. Studies using well-established attachment measures, including the Strange Situation task and the Attachment Q-sort procedure have not consistently linked parental attachment to sleep [156, 157]. Notably, one study found that longer awakenings at 3-months predicted poorer infant-mother attachment at two years, while higher nighttime arousal rates but concurrent longer total sleep duration were linked to stronger infant-father attachment at two years [158]. Additionally, maternal sleep variability has been associated with decreased infant-mother attachment security over time, suggesting a bi-directional relationship within infant-parent dyads [159].

Consistency in bedtime routines and timing also supports healthy sleep by promoting regularity, efficiency, and reduced sleep fragmentation [160, 161]. Globally, bedtime routine implementation varies across countries, with more consistent daily use, defined by parent report, as 4 or more days per week in primarily Caucasian countries compared to primarily Asian countries. Within the United States, the consistent use of bedtime routines is impacted by socioeconomic status, with lower socioeconomic status linked to greater variable sleep, including later bedtimes, more inconsistent routines, and greater nigh-to-night sleep variability [162, 163]. Barriers to consistent sleep patterns often arise from external factors, such as parental work schedules. While consistency in work start times can enhance waketime regularity, late ending or shift work often increases variability in children’s bedtime [161]. Notably, if bedtime is initiated at a time that is misaligned with an infant or toddlers’ underlying intrinsic circadian rhythm, sleep onset latency increases, potentially contributing to sleep onset problems [164]. Circadian rhythms are also influenced by light, with recent research showing that bedtime light exposure in toddlers can cause significant phase delays [165]. Although findings are somewhat mixed, research generally associates lower individual and neighborhood socioeconomic status, disadvantaged environments, and greater neighborhood disorder to adverse sleep outcomes in infants and children [152, 166, 167]. Further studies are needed to examine the direct and indirect effects of these environmental factors on longitudinal changes in sleep.

In children and preadolescents, sleep behavior becomes increasingly influenced by advancing autonomy, school start times, peer and parent relationships, and social media use [40]. With less parental supervision at bedtime, later sleep times and less total sleep duration are often observed [168]. Insufficient sleep during this age period has profound consequences, including poorer emotion regulation, reduced academic achievement, and altered brain morphology [47, 169–171]. In adolescents, decreased sleep duration is associated with increased risk-taking behaviors [172, 173]. Recent research showed that those who reported fewer hours of nighttime sleep had higher odds of mental health symptoms, increased risky driving behaviors, and substance use [172]. Efforts to delay school start times to align with adolescents’ natural circadian rhythms have demonstrated clear benefits, such as improved academic performance and reduced mental health symptoms, risk-taking behaviors, and fewer car accidents [174]. While there is growing social support for increasing weekday sleep duration, the health costs of sleep variability between weekday and weekend, known as social jetlag, remain less recognized. Research suggests that greater social jet lag negatively impacts multiple health domains, including academic performance, cognition, mental health, and emotion regulation [175, 176]. These negative impacts may stem from sleep problems originating in early childhood, which, without early intervention could evolve into persistent sleep-specific problem trajectories [177]. Further, for both children and adolescents, environmental factors are contributing to sleep problems. Although somewhat mixed findings are prevalent, worse sleep is frequently associated with lower socioeconomic status, disadvantaged neighborhoods (i.e. infrastructure disrepair, low social cohesion) with increased exposure to violence, and low perceptions of safety [152, 167, 178]. Research in this area is largely cross-sectional and relies heavily on self-report measures, limiting the ability to assess intra-individual sleep change in relation to socioeconomic or neighborhood factors.

### Adulthood

Sleep behaviors in young and middle adulthood are influenced by several multifaceted and multi-level factors, including interpersonal (e.g., family/caregiving), organizational (e.g., job stress), and environmental (e.g., financial insecurity). Chaos within the home environment, including disorganization, noise, and irregular routines, has been considered an important family-level factor that negatively affects sleep quality in children and parents, particularly those from low SES homes [179]. Perhaps unsurprisingly, mothers are more likely to experience sleep disruptions in relation to their children’s sleep disruptions. In contrast, fathers’ sleep is less strongly associated with their children’s sleep patterns, a finding potentially due to the prevalence of maternal care-taking roles in many areas of the world, including Hong Kong and Iran where these studies were conducted [180, 181]. Indeed, several cross-sectional studies show that the timing, duration, and quality of a child’s sleep are significantly related to the timing, duration, and quality of a mother’s sleep [182], suggesting a potential bi-directional relationship in mother/child dyads for postpartum health outcomes [183]. Such findings highlight the need to consider parenting demands when designing sleep interventions.

Environmental factors, such as work demands, significantly influence sleep health throughout adulthood [184, 185]. Work schedules that disrupt circadian alignments are linked to adverse effects, including inadequate sleep and fatigue [186]. Among these, rotating work shifts present the highest risk for negative outcomes, followed by those with nighttime work schedules [187]. Epidemiological studies suggest that prolonged shift-work not only disrupts sleep but also increases the risk of chronic health conditions, such as cardiovascular disease, diabetes, and metabolic conditions [188, 189]. These findings are especially pertinent given the rising prevalence of shift work, extended work hours, and heightened exposure to artificial light at night due to professional and social obligations [190, 191]. This transition to a 24-hour economy has come at the expense of sleep quality, with significant health consequences [188, 189, 192].

A growing focus in workplace health is presenteeism, which refers to the decline in productivity due to physical or psychosocial conditions while the employee remains at work to remain at work [193, 194]. Presenteeism is recognized as a significant contributor to health-related costs [195]. Factors such as job stress—stemming from high workloads, extended hours, or workplace conflicts—affect productivity and exacerbate presenteeism through its impact on psychological and physical well-being [195]. Notably, research highlights a strong link between presenteeism and sleep disturbances [197–199]. Sleep disturbances impair daytime functioning, lower quality of life, heighten the risk of work-related road accidents, and reduce overall work performance [197, 198]. Studies also indicate a U-shaped relationship between sleep duration and employee health care costs, short-term disability, absenteeism, and presenteeism [198]. Poor sleep quality and insufficient sleep duration are directly associated with higher levels of presenteeism and increased health-related costs [199, 200].

Outside of work, home neighborhood environments can also impact adults’ sleep, with some individuals living in neighborhoods more conducive to a good night of sleep than others. Research shows that individuals residing in socioeconomically disadvantaged neighborhoods as well as urban neighborhoods report lower sleep quality and sleep duration [201, 202]. In the US, members of minoritized groups are more likely to live in neighborhoods with environmental factors that contribute to poorer sleep such as greater light during the night, more noise, and housing with poor indoor temperature control [203]. Along with these environmental disparities that directly impact sleep, it’s suggested that other disparities in disadvantaged neighborhoods such as lack of easy access to affordable and nutritious food along with inadequate recreational spaces for exercise additionally contribute to poor sleep [203]. These compounded social and environmental factors disproportionately affect sleep for those in disadvantaged neighborhoods.

### Older Adults

Psychosocial, physical, cognitive, and socio-environmental factors influence the dynamics of sleep behavior in older adults. One prominent concern is heightened vulnerability to social isolation and loneliness in this demographic cohort [204]. This susceptibility often stems from significant life transitions such as the loss of a partner, solitary living arrangements, dwindling social circles, and a general decline in social engagement [205, 206]. Isolation and loneliness are associated with poorer sleep quality, shorter sleep duration, and increased sleep disruptions [207], with evidence that this relationship is bidirectional [208, 209]. Although depression confounds this relationship, research provides evidence that the link between loneliness and sleep quality persists even after controlling for depression [210]. One theory is that loneliness disrupts sleep via increased difficulty coping with day-to-day stressors, which contributes to maladaptive sleep behaviors [210]. Notably, studies indicate a physiological underpinning to this relationship, as loneliness is linked to increased physiological arousal, as measured by activation of the HPA axis, which regulates glucocorticoid levels and thus modulates the sleep-wake cycle [211]. Subsequent poor sleep may exacerbate feelings of loneliness and diminish an individual’s capability to form connections [208]. For instance, research demonstrates that sleep deprivation heightens sensitivity in brain areas that serve as warnings for human approach, while impairing activity in brain regions which encourage prosociality [212]. Thus, loneliness and poor sleep in older adults maladaptively impact each other.

Age-related decline in cognitive and physical domains introduces additional challenges to maintaining healthy sleep behaviors. The relationship between sleep and age-related cognitive decline is nuanced, with both excessively short (less than 4 h) and long (over 10 h) durations associated with greater decline [213]. Research provides evidence that these extremes in sleep duration coincide with heightened beta-amyloid accumulation, a hallmark of Alzheimer’s pathology as well as a precursor to cognitive decline [214, 215]. Beyond cognitive decline, aging is also associated with a decline in physical activity, accompanied by muscle loss and diminished strength [216]. The reduction in daytime activity may contribute to increased daytime naps in older adulthood due to increased nap opportunity due to more time during the day that is no longer occupied by other activities [217]. Heightened daytime napping is a key shift to sleep patterns experienced in older adulthood though more work is needed to distinguish between adaptive and maladaptive napping behaviors, as discussed further in Sect. 2.2 [72, 218]. Decreased physical activity is also associated with lower quality of sleep. Those with sedentary lifestyles are more prone to poorer sleep and additionally more sensitive to the negative outcomes of poor sleep, such as increased depressive symptoms [219–221]. Across several studies, interventions aimed at increasing physical activity in older adults have shown promising results in promoting both objective and subjective improvements in sleep quality (reviewed in [222]). Thus, initiatives targeting physical activity levels in older adults hold potential in mitigating sleep-related challenges associated with aging.

When older adults experience physical and/or cognitive decline to the point where they cannot adequately care for themselves, they may enter long-term care facilities. These facilities bring about changes in daily routines and environmental factors that can affect sleep behaviors [223–225]. Those in care homes spend more time in monotonous routines (e.g. spending the day going from meal to meal with few structured activities or social interaction), may have roommates with different sleep schedules, and experience disturbances from light during nighttime hours, leading to poor sleep habits [226–228]. For instance, research indicates that older adults are more easily aroused by light disturbances at night, contributing to greater sleep fragmentation and reduced sleep efficiency [229, 230]. This is linked to reduced daytime function and increased daytime sleepiness, which can result in excessive daytime napping as a way to compensate for poor nighttime sleep [231–233]. However, this can create a cycle where excessive naps to compensate for disturbed sleep exacerbate one’s ability to fall asleep and stay asleep [234].

While light during the night is problematic for sleep, insufficient light during the day also leads to sleep complications. Residents of care facilities often experience lower levels of bright light during the day, which can be attributed to lower indoor light levels and limited exposure to natural outdoor light [235]. This lack of exposure to sufficient light, particularly natural daylight, disrupts the body’s circadian rhythm, leading to further sleep disruptions [236]. This is especially harmful to older adults, as they require higher light intensity to synchronize their circadian clock and maintain a normal 24-hour rhythm [74, 237]. Consequently, those residing in care homes with low light levels may be at greater risk for desynchronization. Even outside of care homes, older adults have greater misalignment with light-dark cycles and have a harder time adjusting to jetlag or shift work/night shift (for those still working) [238, 239]. Indeed, research indicates that light therapy can benefit older adults, with improvements to circadian rhythmicity and better sleep outcomes [107, 108].

### Summary

Sleep behaviors are influenced by a variety of factors throughout the lifespan. In infancy, sleep patterns are shaped by factors such as family context, environmental influences, and circadian development. Consistent bedtime routines and alignment with circadian rhythms help promote good sleep, although socio-economic and environmental factors can complicate sleep regularity. As children enter adolescence, sleep behavior is increasingly affected by autonomy, social relationships, and school schedules, with later school start times tied to better sleep and cognitive outcomes. In adulthood, factors such as work demands and family responsibilities additionally influence sleep. Shift work and irregular work hours are particularly detrimental, contributing to poor sleep and increased health risks. As individuals enter older adulthood, physical and/or cognitive decline along with changing living situations increases challenges to maintaining healthy sleep habits. Given the complexity of sleep behaviors across the lifespan, more research is needed to better understand how these behaviors interplay with socio-economic, environmental, and physiological factors. Increased within-subject longitudinal studies are needed in order to draw causal conclusions and assess how these associations develop over time. Disentangling the role of sleep behaviors in health across different life stages can lead to the identification of proactive interventions to promote better sleep behaviors and mitigate associated health outcomes.

## Conclusion

Sleep undergoes dynamic changes in patterns and neurophysiology across the lifespan. Endogenous and exogenous factors play a role in modulating these patterns, influencing behaviors that support or hinder the ability to obtain healthy sleep. The concurrence of changes in sleep physiology and maturation in cortical structures, neurophysiology, hormones, emotion processing, and cognition highlight the crucial mechanistic roles sleep plays. This suggests that sleep’s malleable properties can be effectively leveraged to improve health conditions and enhance quality of life. However, in order to identify interventions most likely to produce sustained effects–since sleep’s impact on health is most effective over long-time scales–the field must expand beyond investigating individual dimensions of sleep at a given age point. While exemplary within-subject longitudinal studies offer valuable insights, such as those by Feinberg, Campbell who tracked sleep electrophysiology biannually across development, such research remains less common [11, 12]. These types of studies, while promising, face challenges such as costs, time constraints, and participant retention over extended periods. Careful planning is essential to address these challenges, including implementation of regular follow-ups, appropriate compensation, and flexible data collection methods (e.g. components which can be completed remotely). Additionally, statistical methods capable of handling missing data can allow researchers to complete robust analyses on all collected data. Accounting for these factors is critical for maximizing the potential of within-subject longitudinal studies.

Well-defined studies focusing on each one of these sleep dimensions individually are essential to move our field forward [8]. As sleep patterns, behaviors and physiology all impact outcomes in health and cognition, the mechanisms by which they do so all ought to be understood in detail on their own. However, we propose a broader vision is necessary for the field to advance past descriptors and into the domain of mechanistic insights: longitudinal burst designed studies that capture quantifiers across all three sleep domains in relation to age, cognition, and health. This approach, which allows for direct identification of causal effects, is vital for designing effective interventions and improving public well-being through a deeper understanding of the complex relationship between sleep, aging, and health.

## Key References

1. Spencer RM, Riggins T. Contributions of memory and brain development to the bioregulation of naps and nap transitions in early childhood. Proc Natl Acad Sci USA. 2021-23415RR.

- These authors theorize that the timing of nap cessation is linked with maturation of the medial temporal lobe network.

2. Tham EKH, Xu H-Y, Fu X, Schneider N, Goh DYT, Lek N, et al. Variations in longitudinal sleep duration trajectories from infancy to early childhood. Sleep Health. 2021;7:56–64.

- These authors longitudinally tracked infants through early childhood and determined sleep clustered into multiple nocturnal trajectories.

3. Nicholson L, Bohnert AM, Crowley SJ. A developmental perspective on sleep consistency: Preschool age through emerging adulthood. Behavioral Sleep Medicine. 2023;21:97–116.

- Excellent review article on sleep consistency from toddlerhood through adulthood, an area often overlooked in research.

4. Grandner MA. Sleep, Health, and Society. Sleep Medicine Clinics. 2022;17:117–39.

- Review article on adverse outcome of sleep and sleep disorders in relation to societal influences.

5. Winer JR, Deters KD, Kennedy G, Jin M, Goldstein-Piekarski A, Poston KL, et al. Association of Short and Long Sleep Duration With Amyloid-β Burden and Cognition in Aging. JAMA Neurology. 2021;78:1187–96.

- The authors demonstrate the negative consequences of short and long sleep in older adults.

6. Burns AC, Saxena R, Vetter C, Phillips AJK, Lane JM, Cain SW. Time spent in outdoor light is associated with mood, sleep, and circadian rhythm-related outcomes: A cross-sectional and longitudinal study in over 400,000 UK Biobank participants. Journal of Affective Disorders. 2021;295:347–52.

- A large, cross-sectional study demonstrating the impact of light on mood disorders and sleep behaviors.

7. Kwon H, Walsh KG, Berja ED, Manoach DS, Eden UT, Kramer MA, et al. Sleep spindles in the healthy brain from birth through 18 years. Sleep. 2023;46:zsad017.

- This paper characterizes age-associated changes in sleep spindles across the first two decades of life.

8. André C, Champetier P, Rehel S, Kuhn E, Touron E, Ourry V, et al. Rapid Eye Movement Sleep, Neurodegeneration, and Amyloid Deposition in Aging. Annals of Neurology. 2023;93:979–90.

- One of the first papers demonstrating changes in REM sleep microstructure are related to greater neurodegeneration.

## Data Availability

No datasets were generated or analysed during the current study.
